# Genome-wide association studies for immunoglobulin concentrations in colostrum and serum in Chinese Holstein

**DOI:** 10.1186/s12864-021-08250-5

**Published:** 2022-01-10

**Authors:** Shan Lin, Cuncun Ke, Lin Liu, Yahui Gao, Lingna Xu, Bo Han, Yaofeng Zhao, Shengli Zhang, Dongxiao Sun

**Affiliations:** 1 Department of Animal Genetics and Breeding, College of Animal Science and Technology, Key Laboratory of Animal Genetics, Breeding and Reproduction of Ministry of Agriculture and Rural Affairs, National Engineering Laboratory for Animal Breeding, China Agricultural University, Beijing, 100193 China; 2 State Key Laboratory of Agrobiotechnology, College of Biological Sciences, China Agricultural University, 100193 Beijing, China; 3 Beijing Dairy Cattle Center, Beijing, 100192 China

**Keywords:** Genome-wide association study, Immunoglobulins, SNP, Immune capacity, Chinese Holstein

## Abstract

**Background:**

The early death and health problems of calves caused substantial economic losses in the dairy industry. As the immune system of neonates has not been fully developed, the absorption of maternal immunoglobulin (Ig) from colostrum is essential in protecting newborn calves against common disease organisms in their early life. The overwhelming majority of Ig in bovine whey is transported from the serum. Therefore, Ig concentration in the colostrum and serum of dairy cows are critical traits when estimating the potential disease resistance of its offspring.

**Results:**

Colostrum, blood, and hair follicle samples were collected from 588 Chinese Holstein cows within 24 h after calving. The concentration of total IgG, IgG1, IgG2, IgA and IgM in both colostrum and serum were detected via ELISA methods. With GCTA software, genome-wide association studies (GWASs) were performed with 91,620 SNPs genotyped by GeneSeek 150 K (140,668 SNPs) chips. As a result, 1, 5, 1 and 29 significant SNPs were detected associated with the concentrations of colostrum IgG1, IgG2, IgA IgM, and serum IgG2 at the genome-wide level (*P* < 3.08E–6); 11, 2, 13, 2, 12, 8, 2, 27, 1 and 4 SNPs were found significantly associated with total IgG, IgG1, IgG2, IgA and IgM in colostrum and serum at the suggestive level (*P* < 6.15E–5). Such SNPs located in or proximate to (±1 Mb) 423 genes, which were functionally implicated in biological processes and pathways, such as immune response, B cell activation, inflammatory response and NF-kappaB signaling pathways. By combining the biological functions and the known QTL data for immune traits in bovine, 14 promising candidate functional genes were identified for immunoglobulin concentrations in colostrum and serum in dairy cattle, they were *FGFR4*, *FGFR2*, *NCF1*, *IKBKG*, *SORBS3*, *IGHV1S18*, *KIT*, *PTGS2*, *BAX*, *GRB2*, *TAOK1*, *ICAM1*, *TGFB1* and *RAC3.*

**Conclusions:**

In this study, we identified 14 candidate genes related to concentrations of immunoglobulins in colostrum and serum in dairy cattle by performing GWASs. Our findings provide a groundwork for unraveling the key genes and causal mutations affecting immunoglobulin concentrations in colostrum and important information for genetic improvement of such traits in dairy cattle.

**Supplementary Information:**

The online version contains supplementary material available at 10.1186/s12864-021-08250-5.

## Background

The early survival rate and health of calves are important factors affecting the production efficiency of the dairy industry. It was reported that approximately 31% of preweaning mortality events occurring in the first 3 weeks of life were attributed to the low serum IgG concentration of calf (less than 10 mg/mL when sampled between 24 and 48 h of age) [1, 2]. Indeed, the immune system of neonates has not been fully developed depending almost entirely on the transport of maternal immunoglobulin (Ig) from colostrum after birth. Hence, the absorption of colostrum Ig during the first 24 h after birth is essential for the health and survival of the neonatal calf.

Immunoglobulins are the major protein components of colostrum, comprising 70–80% of the total protein content, while in mature milk, immunoglobulins constitute only 1–2% [3, 4]. There are three major immunoglobulins in bovine serum and milk: IgG, IgM and IgA, with IgG consisting of two subclasses (IgG1 and IgG2). IgG1 accounts for over 75% (46.4 mg/ml), and IgM (6.8 mg/ml), IgA (5.4 mg/ml) and IgG2 (2.9 mg/ml) are followed successively [3]. Immunoglobulins are produced by B1-cells and possess a multitude of functions such as activate complement-mediated bacteriolytic reactions, augment the recognition and phagocytosis of bacteria by leucocytes (opsonization), prevent the adhesion of microbes to surfaces, inhibit bacterial metabolism, agglutinate bacteria, and neutralize toxins and viruses [5]. Bovine colostrum immunoglobulins are notably transported from the serum and accumulated in the mammary gland during the prepartum dry period [6, 7]. Hence, delineation of the genetic architecture underlying the concentrations of immunoglobulins in cows’ colostrum and serum is important for identifying ways to improve the survival rate of neonatal calves in dairy cattle.

Concentrations of immunoglobulins are typical quantitative characteristics controlled by multiple QTLs and polygenes [8]. Heritability estimates for IgG concentrations in blood ranged from 0.27 to 0.64 in humans [9–12]. Similarly, heritability estimates of immunoglobulins in serum and milk in dairy cattle ranged from 0.08 to 0.45 [13–16]. Heritability for IgM was higher than IgG ranging from 0.18 to 0.45 and from 0.08 to 0.23, respectively. Estimates of heritability in serum were generally higher than in milk (0.15–0.25) [13].

Genome-wide association studies (GWASs) have been performed for immunoglobulins in serum or mature milk. The first GWAS based on 2247 individuals from four European cohorts (CROATIA-Vis, CROATIA-Korcula, Orkney Complex Disease Study and Northern Swedish Population Health Study) identified 9 genome-wide significant loci associate with IgG glycosylation and 4 out of them contained genes encoding glycosyltransferases [17]. Another GWAS for IgG glycosylation patterns in humans indicated that RUNX family transcription factor 3 (RUNX3) was associated with decreased galactosylation and involved in both IgA class switching and B-cell maturation as well as T-cell differentiation and apoptosis [18]. In pigs, 2 genome-wide and 4 chromosome-wide significant SNPs were detected for IgG blocking percentage to CSF virus in serum by performing GWAS [19]. Especially, a GWAS for blood natural antibodies in Canadian Holstein cows identified 23 SNPs that were significantly associated with IgG concentration at genome-wide level [20]. Another GWAS for milk natural antibodies in Dutch Holstein-Friesian cattle identified some significant SNPs for IgG1 and IgM with candidate genes on *Bos taurus* autosome (BTA) 3, 17, 18, and 21 that related to immunoglobulin structure and early B cell development [21]. However, there are few studies on gene identification for immunoglobulin concentrations in colostrum in dairy cattle so far. In addition, in bovine colostrum, the immunoglobulins were found mainly derived from serum [6, 7]. Hence, investigation on the concentrations of immunoglobulins in both colostrum and serum can better disentangle the genetic architecture underlying colostrum immunoglobulin traits. Here, we conducted genome-wide association studies for the concentrations of immunoglobulin components in colostrum and serum in a Chinese Holstein population to identify the functional genes that contributed to the phenotypic variation of colostrum immunoglobulins and provide molecular information for genetically improving such traits to increase calves’ disease-resistance.

## Results

### Statistics of phenotypes

In this study, we measured the concentrations of immunoglobulins in both colostrum and serum for 588 Chinese Holstein cows. As a result, a total of 10 traits were recorded, including concentrations of total IgG, IgG1, IgG2, IgA and IgM. Means and the corresponding standard deviations for the original and corrected phenotypic values were shown in Table 1. The estimated heritability of total IgG, IgG1, IgG2, IgA and IgM concentrations in colostrum and serum ranged from was at 0.094 to 0.48 and from 0.087 to 0.295, respectively (Table 2).

**Table 1:** Means and standard deviations for the original and corrected concentrations of immunoglobulins in colostrum and serum (*N* = 588)

Traits	Original				Transformed			
	Mean (mg/ml)	SD	Min	Max	Mean	SD	Min	Max
col_IgG	33.46	43.64	1.16	342.05	5.16	2.62	1.08	18.49
col_IgG1	14.89	7.64	0.24	44.83	3.72	1.04	0.49	6.7
col_IgG2	3.16	1.93	0.07	11.04	1.66	0.59	0.26	3.32
col_IgA	3.07	3.65	0.01	51.18	0.27	0.49	−1.91	1.71
col_IgM	5.31	3.38	0.09	20.86	0.62	0.35	−1.06	1.32
ser_IgG	8.36	3.34	0.68	22.9	2.84	0.57	0.82	4.79
ser_IgG1	1.03	0.7	0.01	5.52	0.97	0.31	0.09	2.35
ser_IgG2	13.68	6.26	0.69	42.36	3.6	0.83	0.83	6.51
ser_IgA	0.23	0.16	0.01	1.17	−0.74	0.4	−5.4	0.07
ser_IgM	2.25	1.71	0.01	14.93	0.24	0.35	−2.34	1.17

*N* sample number, *Mean* arithmetic mean, *SD* standard deviation, *Min* minimum, *Max* maximum, *col_IgG* col_IgG1, col_IgG2, col_IgA and col_IgM represented the concentration of total IgG, IgG1, IgG2, IgA and IgM in colostrum, respectively; ser_IgG, ser_IgG1, ser_IgG2, ser_IgA and ser_IgM represented the concentration of total IgG, IgG1, IgG2, IgA and IgM in serum, respectively

**Table 2:** The estimated heritability of concentrations of immunoglobulins in colostrum and serum

Traits	Colostrum	Serum
IgG	0.235 ± 0.069	0.141 ± 0.078
IgG1	0.125 ± 0.070	0.078 ± 0.084
IgG2	0.094 ± 0.067	0.087 ± 0.070
IgA	0.329 ± 0.083	0.295 ± 0.085
IgM	0.482 ± 0.092	0.206 ± 0.087

### Genome-wide association study

**Fig. 1:**
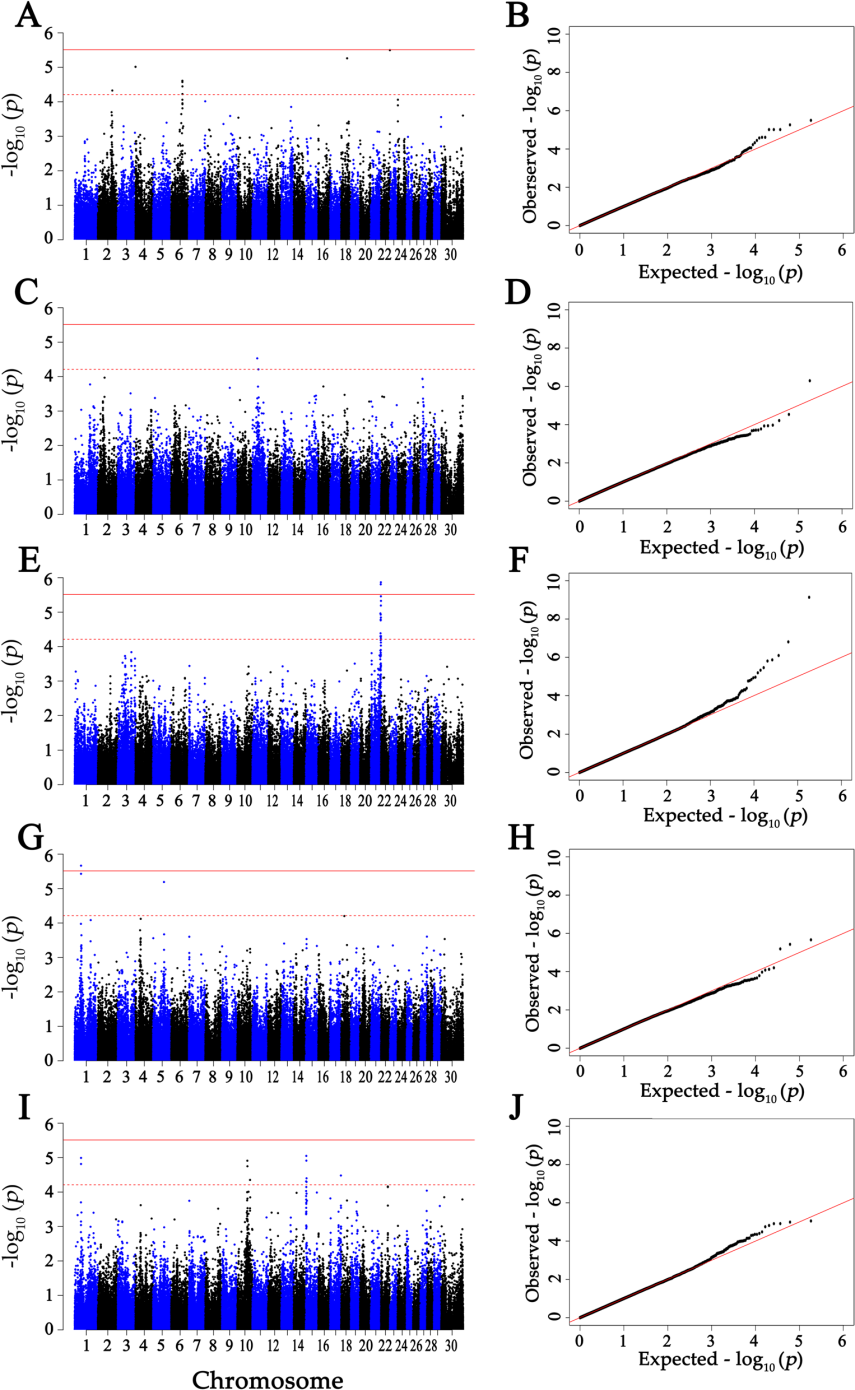
Manhattan and Q-Q plots of the observed *P*-values for the concentrations of immunoglobulins in the colostrum. **A** and **B** Indicated IgG concentrations. **C** and **D** Indicated IgG1 concentrations. **E** and **F** Indicated IgG2 concentrations. **G** and **H** Indicated IgA concentrations. **I** and **J** Indicated IgM concentrations. The Manhattan plots presented −log10 (*P*-values) for genome-wide SNPs (y-axis) plotted against their respective positions on each chromosome (x-axis), the horizontal red and red dashed lines in the Manhattan plots indicated the genome-wide (3.08E–6) and suggestive significance (6.15E–5) thresholds, respectively. The Q-Q plots showed the observed −log10-transformed *P*-values (y-axis) and the expected −log10-transformed *P*-values (x-axis)

**Fig. 2:**
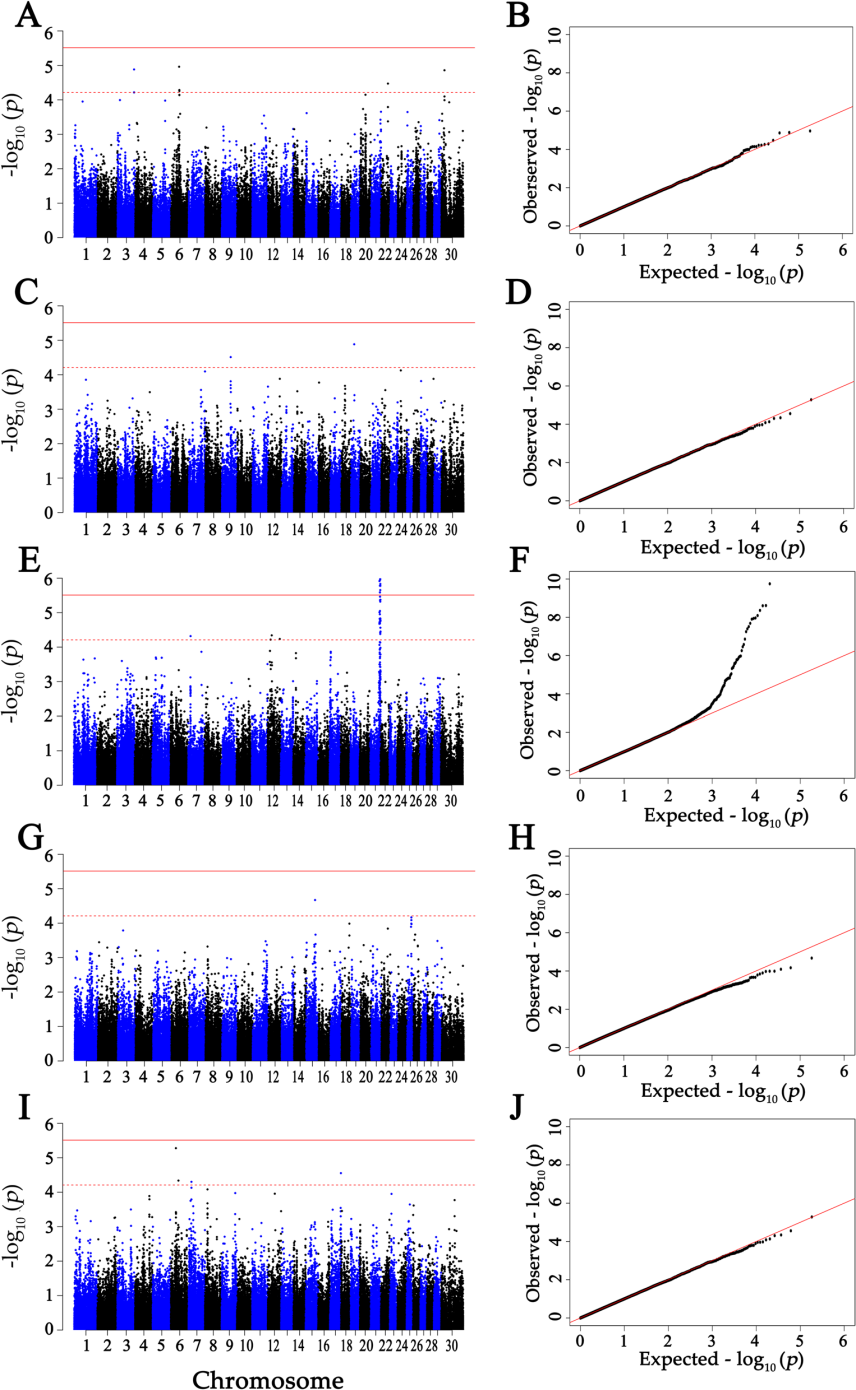
Manhattan and Q-Q plots of the observed *P*-values for the concentrations of immunoglobulins in the serum. **A** and **B** Indicated IgG concentrations. **C** and **D** Indicated IgG1 concentrations. **E** and **F** Indicated IgG2 concentrations. **G** and **H** Indicated IgA concentrations. **I** and **J** Indicated IgM concentrations

After LD analysis, a total of 16,257 effectively independent tests number were suggested. Thus, the threshold *P*-value for genome-wide significant association was set at 3.08E–6 (0.05/16,257) and that for suggestive significant association was 6.15E–5 (1/16,257) [22]. Based on the QQ plots (Figs. 1 and 2) and the estimated inflation factor (λ) of 0.98–1.03 for all traits, no population stratification was observed.

With GCTA 1.90.2, we performed the GWASs for the 10 traits. In colostrum (Fig. 1 and Table 3), significant associations between 11 SNPs and total IgG were found at the suggestive level (*P* < 6.15E–5). The significant SNPs were located on BTA 2 (1 SNP), 4 (3 SNPs), 6 (5 SNPs), 18 (1 SNP), 22 (1 SNP). For IgG1, one genome-wide significant SNP (*P* < 3.08E-6) on BTA 13 and two suggestive significant SNPs (*P* < 6.15E–5) on BTA 11 were detected. For IgG2, five genome-wide significant SNPs (*P* < 3.08E-6) and 13 suggestive significant SNPs (*P* < 6.15E–5) were observed, locating on BTA 20 (1 SNP) and 21 (17 SNPs). There was one genome-wide significant SNP (*P* < 3.08E-6) and two suggestive significant SNPs (*P* < 6.15E–5) detected associated with IgA, locating on BTA 5 (1 SNP) and 11 (2 SNPs). Twelve SNPs were significantly associated with IgM at the suggestive level (*P* < 6.15E–5), distributing on BTA 1 (2 SNPs), 10 (4 SNPs), 15 (5 SNPs) and 17 (1 SNP).

**Table 3:** The significant SNPs for concentrations of total IgG, IgG1, IgG2, IgA and IgM in colostrum

Traits^a^	Chr^b^	SNP name	Position (bp)	Major/minor allele	MAF^c^	SNP effect	SE^d^	*P*-value
col_IgG	2	BovineHD0200028482	98,947,581	G/A	0.36	−12.63	3.10	4.70E-05
col_IgG	4	ARS-BFGL-NGS-67542	190,619	A/G	0.38	13.20	2.98	9.66E-06
col_IgG	4	ARS-BFGL-NGS-114472	253,919	C/A	0.38	13.18	2.98	9.66E-06
col_IgG	4	ARS-BFGL-NGS-102812	360,794	G/A	0.38	13.18	2.98	9.66E-06
col_IgG	6	BovineHD0600021193	76,307,745	A/G	0.29	14.03	3.34	2.66E-05
col_IgG	6	BovineHD0600021227	76,365,244	G/A	0.29	14.08	3.34	2.45E-05
col_IgG	6	BovineHD0600021233	76,396,527	A/G	0.29	14.08	3.34	2.45E-05
col_IgG	6	Hapmap50522-BTA-98140	76,718,463	A/G	0.40	12.50	3.11	5.94E-05
col_IgG	6	BovineHD0600021300	76,762,984	A/C	0.41	12.70	3.07	3.57E-05
col_IgG	18	ARS-BFGL-NGS-88483	40,843,328	A/G	0.49	−13.35	2.94	5.47E-06
col_IgG	22	BovineHD2200017739	60,902,629	A/C	0.24	15.87	3.41	3.18E-06
col_IgG1	11	ARS-BFGL-NGS-27262	32,766,825	A/T	0.35	−0.31	0.07	2.94E-05
col_IgG1	11	BovineHD1100011957	40,807,680	A/G	0.35	−0.30	0.07	6.15E-05
col_IgG1	13	ARS-BFGL-NGS-11585	17,407,043	C/A	0.34	−0.39	0.08	5.10E-07
col_IgG2	20	BovineHD2000021006	71,896,856	G/A	0.27	0.24	0.04	7.30E-10
col_IgG2	21	BovineHD2100019814	67,542,721	A/C	0.36	0.16	0.04	1.09E-05
col_IgG2	21	BovineHD4100015366	67,786,313	A/G	0.42	0.14	0.04	4.99E-05
col_IgG2	21	ARS-BFGL-NGS-86477	68,399,787	A/C	0.48	−0.14	0.04	4.11E-05
col_IgG2	21	BovineHD2100020225	69,289,258	A/G	0.33	0.15	0.04	1.64E-05
col_IgG2	21	BovineHD2100020241	69,357,379	A/C	0.42	0.16	0.04	1.18E-05
col_IgG2	21	ARS-BFGL-NGS-115062	69,395,154	G/A	0.44	0.15	0.04	1.73E-05
col_IgG2	21	BovineHD2100020341	69,673,486	A/G	0.36	0.17	0.03	1.56E-06
col_IgG2	21	BovineHD2100020413	69,920,970	G/A	0.38	0.14	0.03	5.64E-05
col_IgG2	21	BovineHD2100020670	70,592,463	A/C	0.41	0.14	0.03	5.65E-05
col_IgG2	21	ARS-BFGL-NGS-2644	70,608,408	A/G	0.35	0.18	0.04	1.36E-06
col_IgG2	21	BovineHD2100020676	70,621,565	G/A	0.35	0.18	0.04	8.02E-07
col_IgG2	21	BovineHD2100020685	70,655,075	A/G	0.32	0.20	0.04	1.56E-07
col_IgG2	21	BovineHD2100020689	70,672,433	A/G	0.38	0.16	0.04	6.43E-06
col_IgG2	21	BovineHD2100020696	70,687,439	G/A	0.37	0.15	0.03	1.43E-05
col_IgG2	21	ARS-BFGL-NGS-73522	70,702,245	G/A	0.35	0.17	0.04	3.47E-06
col_IgG2	21	BovineHD2100020833	71,318,798	A/G	0.29	0.17	0.04	4.71E-06
col_IgG2	21	BovineHD2100020853	71,389,313	G/A	0.25	−0.17	0.04	4.96E-05
col_IgA	1	BovineHD0100012272	43,118,172	C/A	0.43	0.16	0.03	3.73E-06
col_IgA	1	BovineHD0100012276	43,138,880	C/A	0.43	0.16	0.03	2.16E-06
col_IgA	5	BovineHD0500021305	74,998,613	G/A	0.34	−0.15	0.03	6.46E-06
col_IgM	1	BovineHD0100012272	43,118,172	C/A	0.43	0.10	0.02	1.53E-05
col_IgM	1	BovineHD0100012276	43,138,880	C/A	0.43	0.10	0.02	1.02E-05
col_IgM	10	BovineHD1000019825	69,080,826	C/A	0.15	−0.13	0.03	1.23E-05
col_IgM	10	BovineHD1000019983	69,718,494	A/C	0.31	−0.10	0.02	1.78E-05
col_IgM	10	BovineHD1000024944	87,623,949	A/G	0.44	0.09	0.02	4.44E-05
col_IgM	10	BovineHD1000024954	87,671,849	A/G	0.44	0.09	0.02	4.44E-05
col_IgM	15	BovineHD1500000388	1,498,954	G/A	0.49	0.09	0.02	4.89E-05
col_IgM	15	BovineHD1500000626	2,664,438	A/G	0.48	−0.10	0.02	8.90E-06
col_IgM	15	BovineHD1500000698	3,061,918	A/G	0.35	−0.10	0.02	1.22E-05
col_IgM	15	BTB-01900838	4,285,360	A/G	0.32	0.10	0.02	3.98E-05
col_IgM	15	BovineHD1500001009	4,395,956	G/A	0.31	0.09	0.02	5.07E-05
col_IgM	17	ARS-BFGL-NGS-117653	73,315,120	A/C	0.40	−0.09	0.02	3.30E-05

^a^col_IgG, col_IgG1, col_IgG2, col_IgA and col_IgM represented concentration of total IgG, IgG1 IgG2, IgA and IgM in colostrum, respectively

^b^Cow chromosome number

^c^Minor allele frequency

^d^standard error

In serum (Fig. 2 and Table 4), eight SNPs were found significantly associated with total IgG at the suggestive level (*P* < 6.15E–5), locating on BTA 3 (3 SNPs), 6 (3 SNPs), 22 (1 SNP) and 30 (1 SNP). Two SNPs located on BTA 9 and 19 had significant associations with total IgG1 at the suggestive level (*P* < 6.15E–5). For IgG2, 29 genome-wide significant SNPs (*P* < 3.08E-6) and 27 suggestive significant SNPs (*P* < 6.15E–5) were detected, locating on 7 (1 SNP), 12 (3 SNPs), BTA 20 (1 SNP) and 21 (51 SNPs). Additionally, one and four SNPs were significantly associated with the concentration of IgA and IgM at the suggestive level (*P* < 6.15E–5), respectively. Significant SNPs were located on BTA 15 (1 SNP), BTA6 (2 SNPs), 7 (1 SNP) and 17 (1 SNP).

**Table 4:** The significant SNPs for concentrations of total IgG, IgG1, IgG2, IgA and IgM in serum

Traits^a^	Chr^b^	SNP name	Position (bp)	Major/minor allele	MAF^c^	SNP effect	SEd	*P*-value
ser_IgG	3	BovineHD0300032641	112,978,010	A/C	0.20	−0.18	0.04	6.12E-05
ser_IgG	3	BovineHD0300032649	113,013,960	A/G	0.20	−0.18	0.04	6.06E-05
ser_IgG	3	BovineHD0300032659	113,046,130	G/A	0.18	−0.20	0.05	1.31E-05
ser_IgG	6	ARS-BFGL-NGS-100863	54,826,213	G/A	0.26	0.17	0.04	1.10E-05
ser_IgG	6	BovineHD0600015431	56,407,206	G/A	0.21	0.17	0.04	5.20E-05
ser_IgG	6	BovineHD0600015455	56,506,982	G/A	0.20	0.17	0.04	5.55E-05
ser_IgG	22	ARS-BFGL-NGS-111049	46,902,036	G/A	0.35	0.15	0.04	3.38E-05
ser_IgG	30	BovineHD3000006683	20,390,212	A/G	0.40	0.16	0.04	1.39E-05
ser_IgG1	9	BovineHD0900016412	59,785,726	G/A	0.26	0.10	0.02	3.08E-05
ser_IgG1	19	BovineHD1900006278	21,930,663	A/G	0.27	−0.10	0.02	1.31E-05
ser_IgG2	7	BovineHD0700004317	15,718,585	G/A	0.47	0.20	0.05	4.79E-05
ser_IgG2	12	BovineHD1200006401	21,324,076	G/A	0.37	0.22	0.06	6.06E-05
ser_IgG2	12	BovineHD1200008568	28,980,452	C/A	0.34	0.24	0.06	4.55E-05
ser_IgG2	12	BovineHD1200024367	84,101,237	A/G	0.48	0.21	0.05	5.80E-05
ser_IgG2	20	BovineHD2000021006	71,896,856	G/A	0.28	0.47	0.06	1.01E-14
ser_IgG2	21	BovineHD2100018564	63,334,736	A/C	0.41	−0.24	0.06	2.10E-05
ser_IgG2	21	BovineHD2100018787	63,926,754	G/A	0.47	−0.26	0.05	1.21E-06
ser_IgG2	21	BovineHD2100018795	63,954,059	G/A	0.35	0.24	0.06	1.89E-05
ser_IgG2	21	BTA-24891-no-rs	63,955,841	G/A	0.36	0.24	0.05	9.73E-06
ser_IgG2	21	BovineHD2100019235	65,578,768	A/G	0.48	−0.23	0.05	1.49E-05
ser_IgG2	21	BovineHD2100019547	66,578,213	A/G	0.40	−0.24	0.05	1.11E-05
ser_IgG2	21	BovineHD2100019656	66,910,728	G/A	0.42	0.28	0.05	2.64E-07
ser_IgG2	21	BovineHD2100019670	66,973,587	A/C	0.39	0.28	0.06	5.53E-07
ser_IgG2	21	ARS-BFGL-NGS-37313	66,988,787	C/A	0.40	0.26	0.05	2.58E-06
ser_IgG2	21	BovineHD2100019681	67,009,668	A/G	0.40	0.26	0.05	1.65E-06
ser_IgG2	21	ARS-BFGL-NGS-107488	67,030,857	A/G	0.31	−0.24	0.06	2.07E-05
ser_IgG2	21	ARS-BFGL-NGS-20339	67,088,847	G/A	0.33	0.26	0.06	2.57E-06
ser_IgG2	21	BovineHD2100019763	67,342,472	C/A	0.44	0.25	0.06	8.91E-06
ser_IgG2	21	BovineHD2100019814	67,542,721	A/C	0.36	0.31	0.06	3.08E-08
ser_IgG2	21	BovineHD2100019834	67,604,077	A/C	0.15	0.33	0.08	1.60E-05
ser_IgG2	21	BovineHD2100019854	67,706,221	G/A	0.30	−0.25	0.06	1.85E-05
ser_IgG2	21	BovineHD4100015366	67,786,313	A/G	0.42	0.31	0.06	2.07E-08
ser_IgG2	21	BovineHD2100019888	67,885,290	A/G	0.21	0.29	0.06	4.33E-06
ser_IgG2	21	BovineHD2100019906	67,946,189	A/G	0.32	0.27	0.06	3.43E-06
ser_IgG2	21	ARS-BFGL-NGS-86477	68,399,787	A/C	0.48	−0.27	0.06	1.11E-06
ser_IgG2	21	BovineHD2100020097	68,740,864	G/A	0.30	0.25	0.06	1.68E-05
ser_IgG2	21	BovineHD2100020157	69,009,950	A/C	0.44	0.27	0.05	1.05E-06
ser_IgG2	21	BovineHD2100021033	69,033,145	G/A	0.20	0.38	0.07	8.11E-09
ser_IgG2	21	BovineHD2100020203	69,206,894	G/A	0.33	−0.23	0.06	3.77E-05
ser_IgG2	21	BovineHD2100020225	69,289,258	A/G	0.33	0.31	0.05	1.15E-08
ser_IgG2	21	BovineHD2100020232	69,327,116	G/A	0.35	0.32	0.05	4.36E-09
ser_IgG2	21	BovineHD2100020241	69,357,379	A/C	0.42	0.25	0.06	4.78E-06
ser_IgG2	21	ARS-BFGL-NGS-115062	69,395,154	G/A	0.44	0.26	0.06	2.21E-06
ser_IgG2	21	BovineHD2100020269	69,440,566	A/G	0.29	−0.25	0.06	4.16E-05
ser_IgG2	21	BovineHD2100020314	69,587,749	G/A	0.36	−0.24	0.05	1.51E-05
ser_IgG2	21	BovineHD2100020317	69,613,677	G/A	0.24	0.28	0.06	1.47E-05
ser_IgG2	21	BovineHD2100020325	69,637,166	A/G	0.36	−0.23	0.05	3.79E-05
ser_IgG2	21	BovineHD2100020341	69,673,486	A/G	0.36	0.34	0.05	1.78E-10
ser_IgG2	21	BovineHD2100020413	69,920,970	G/A	0.38	0.30	0.05	5.65E-08
ser_IgG2	21	ARS-BFGL-NGS-1345	69,939,350	C/A	0.42	0.26	0.05	1.47E-06
ser_IgG2	21	BovineHD2100020425	69,955,674	G/A	0.45	0.24	0.05	1.55E-05
ser_IgG2	21	BovineHD2100020439	70,000,656	A/G	0.45	0.27	0.06	1.66E-06
ser_IgG2	21	ARS-USDA-AGIL-chr21–70,182,028-000470	70,182,028	C/G	0.38	0.23	0.05	2.74E-05
ser_IgG2	21	BovineHD2100020583	70,430,736	G/A	0.23	0.29	0.06	4.23E-06
ser_IgG2	21	BovineHD2100020653	70,537,404	A/G	0.18	0.38	0.07	1.09E-08
ser_IgG2	21	BovineHD2100020670	70,592,463	A/C	0.41	0.29	0.05	1.38E-07
ser_IgG2	21	ARS-BFGL-NGS-2644	70,608,408	A/G	0.35	0.38	0.06	3.24E-11
ser_IgG2	21	BovineHD2100020676	70,621,565	G/A	0.35	0.39	0.06	8.11E-12
ser_IgG2	21	BovineHD2100020685	70,655,075	A/G	0.32	0.39	0.06	2.23E-11
ser_IgG2	21	BovineHD2100020689	70,672,433	A/G	0.38	0.29	0.06	3.46E-07
ser_IgG2	21	BovineHD2100020696	70,687,439	G/A	0.37	0.32	0.05	2.50E-09
ser_IgG2	21	ARS-BFGL-NGS-73522	70,702,245	G/A	0.35	0.32	0.06	1.25E-08
ser_IgG2	21	Hapmap54369-rs29015082	71,109,676	A/G	0.45	−0.22	0.05	3.52E-05
ser_IgG2	21	BovineHD2100020833	71,318,798	A/G	0.29	0.32	0.06	4.01E-08
ser_IgG2	21	BovineHD2100020847	71,359,883	A/G	0.14	0.33	0.08	1.72E-05
ser_IgG2	21	BovineHD2100020883	71,479,429	A/G	0.16	0.43	0.07	2.43E-09
ser_IgA	15	BTA-91367-no-rs	60,316,301	A/G	0.47	−0.11	0.03	2.13E-05
ser_IgM	6	BovineHD0600009523	34,015,077	G/A	0.28	−0.11	0.02	5.25E-06
ser_IgM	6	BovineHD0600014163	51,369,747	G/A	0.12	−0.14	0.03	4.59E-05
ser_IgM	7	ARS-BFGL-NGS-12159	19,220,954	A/C	0.44	−0.09	0.02	4.98E-05
ser_IgM	17	BovineHD1700021706	74,223,510	G/A	0.17	−0.12	0.03	2.79E-05

^a^ser_IgG, ser_IgG1, ser_IgG2, ser_IgA and ser_IgM represented concentration of total IgG, IgG1 IgG2, IgA and IgM in serum, respectively

^b^Cow Chromosome number

^c^Minor allele frequency

^d^standard error

### Candidate genes and function analysis

After comparing to the reference genes (UMD 3.1), a total of 423 genes that contained or were adjacent to (± 1 Mb) the significant SNPs were mapped, including 392 protein-coding genes, 30 non-coding RNAs and 1 pseudogene (Additional file 1: Table S1).

To further investigate the biological functions of these candidate genes, we performed GO and KEGG analysis and observed that 73 genes were enriched in immune-related biological processes and pathways such as adaptive immune response based on somatic recombination of immune receptors built from immunoglobulin superfamily domains, immune response, B cell activation, inflammatory response, and NF-kappaB signaling pathways (Additional file 2: Table S2). Simultaneously, we compared the physical positions of the 423 genes with the peak of the known QTLs that have been shown associated with immune capacity in dairy cattle (Cattle QTLdb), including IgG level, FMDV peptide-induced cell proliferation, ConA-induced cell proliferation and Clinical mastitis. Consequently, 226 genes were found located within the QTL regions with a distance to the peak positions of less than 1.0 cM.

Integrating the results of GO/KEGG and QTL data, 14 overlapping genes were considered as promising candidates for the concentrations of immunoglobulins in colostrum and serum (Table 5). They were fibroblast growth factor receptor 4 (*FGFR4*), fibroblast growth factor receptor 2 (*FGFR2*), neutrophil cytosolic factor 1 (*NCF1*), inhibitor of nuclear factor kappa B kinase regulatory subunit gamma (*IKBKG*), sorbin and SH3 domain containing 3 (*SORBS3*), immunoglobulin heavy variable 4–59 *(IGHV1S18*), KIT proto-oncogene, receptor tyrosine kinase (*KIT*), prostaglandin-endoperoxide synthase 2 (*PTGS2*), BCL2 associated X, apoptosis regulator (*BAX*), growth factor receptor bound protein 2 (*GRB2*), Thousand and one kinase 1 (*TAOK1*), intercellular adhesion molecule 1 (*ICAM1*), transforming growth factor beta 1 (*TGFB1*), and Rac family small GTPase 3 (*RAC3*).

**Table 5:** The list of candidate genes contained or nearby the significant SNPs associated with total IgG, IgG1, IgG2, IgA and IgM in the colostrum and serum

Gene ID	Gene Name	Chr^a^	Gene Start^b^	Gene End^b^	Traits
ENSBTAG00000010543	*FGFR4*	18	39,936,163	39,946,911	col_IgG
ENSBTAG00000014064	*FGFR2*	18	41,823,602	41,930,655	col_IgG
ENSBTAG00000003305	*NCF1*	11	33,267,455	33,282,333	col_IgG1
ENSBTAG00000006268	*IKBKG*	11	40,501,901	40,519,263	col_IgG1
ENSBTAG00000014401	*SORBS3*	21	70,357,692	70,384,760	col_IgG2, ser_IgG2
ENSBTAG00000053635	*IGHV1S18*	21	71,529,984	71,530,481	col_IgG2, ser_IgG2
ENSBTAG00000002699	*KIT*	21	71,796,317	71,917,430	col_IgG2, ser_IgG2
ENSBTAG00000014127	*PTGS2*	10	69,263,775	69,271,399	col_IgM
ENSBTAG00000013340	*BAX*	6	55,985,201	55,989,210	ser_IgG
ENSBTAG00000004736	*GRB2*	6	56,754,111	56,818,428	ser_IgG
ENSBTAG00000000827	*TAOK1*	19	21,308,164	21,363,337	ser_IgG1
ENSBTAG00000010303	*ICAM1*	7	16,040,883	16,051,454	ser_IgG2
ENSBTAG00000020457	*TGFB1*	6	50,772,077	50,785,924	ser_IgM
ENSBTAG00000022927	*RAC3*	6	51,470,005	51,471,808	ser_IgM

^a^Cow chromosome number

^b^The position of gene was based on the UMD 3.1 assembly

col_IgG, col_IgG1, col_IgG2, col_IgA and col_IgM represented concentration of total IgG, IgG1 IgG2, IgA and IgM in colostrum; ser_IgG, ser_IgG1, ser_IgG2, ser_IgA and ser_IgM represented concentration of total IgG, IgG1 IgG2, IgA and IgM in serum

## Discussion

In this study, we identified the chromosome regions related with immunoglobulin concentrations in colostrum and serum in dairy cattle by performing GWASs with high density SNP genotypes. Consequently, we detected 19, 5, 74, 4 and 16 significant SNPs associated with the total IgG, IgG1, IgG2, IgA and IgM, respectively. To our knowledge, this is the first investigation on the genetic architecture of colostrum immunoglobulins in dairy cattle.

In general, a genomic inflation factor λ of < 1.05 suggests no population stratification [23]. In this study, the calculated λ values ranged from 0.99 to 1.03 for concentrations of Ig concentration in colostrum and serum, suggesting population stratification was well controlled.

In the present study, the significant SNPs associated with the concentration of IgG2 in colostrum and serum were almost entirely distributed on BTA21 from 63.3 to 71.5 Mb. Similarly, two previous GWASs in Canadian and Dutch Holstein populations observed that the significant SNPs for IgG in serum and IgG1 in mature milk were mainly located in BTA21 from 55.5 to 70.6 Mb and 66.0 to 71.6 Mb, which contained the region identified in our study [20, 21]. The previous studies revealed that the main locus of bovine immunoglobulin heavy chain variable genes was located on approximately 71.5 Mb of BTA21 [24, 25], indicating this region may be related to the formation of immunoglobulin. Concurrently, the significant SNPs for the total IgG and IgG1 concentrations in colostrum and serum distributed on multiple chromosomes, including BTA2, 3, 4, 6, 9, 11, 18, 19, 22 and 30, which is inconsistent with the previous two GWASs for IgG in serum and IgG1 in mature milk. Such inconsistency was most likely due to the huge difference of Ig formation mechanism and concentration between colostrum and mature milk. The majority of bovine colostrum Ig was transported from serum and accumulate in the mammary gland during the prepartum dry period, under the influence of prolactin and ceases abruptly at parturition, resulting in 200 times difference between Ig concentration in colostrum and mature milk [26]. Furthermore, 2 significant SNPs associated with IgM concentrations in colostrum and serum were detected on 73.3 and 74.2 Mb of BTA17, very close to 2 significant SNPs on BTA17 (72.5 to 73.6 Mb) identified for IgM in mature milk in a previous GWAS in Dutch Holstein populations [20]. The remaining significant SNPs were first reported in this study.

Combing the biological functions of the 423 functional genes that contained or were closed to the significant SNPs with less than 1 Mb and the known QTL data for immune traits in bovine,14 promising genes were identified for Ig. Of these, 2, 2, 3 and 1 candidate genes were selected for the total IgG, IgG1, IgG2 and IgM concentration in colostrum, respectively. *FGFR2* and *FGFR4* belong to the fibroblast growth factor receptor family which has been shown to mediate pro-inflammatory signaling in the liver and airway epithelium in chronic obstructive pulmonary disease [27]. *NCF1* encodes a cytosolic subunit of neutrophil NADPH oxidase, an enzyme responsible for reactive oxygen species (ROS) production, which is pivotal in both host defense and the control of inflammation [28, 29]. *IKBKG* encodes the regulatory subunit of the inhibitor of kappaB kinase (IKK) complex, which activates NF-kappaB resulting activation of genes involved in inflammation and immunity [30, 31]. *SORBS3* encodes an SH3 domain-containing adaptor protein that regulates cell adhesion and signal transduction. The deficiency of adaptor protein could suppress vascular inflammation and inactivate Akt–nuclear factor κB signaling [32]. *IGHV1S18* is an immunoglobulin heavy chain variable region that encodes Ig heavy chain and is directly related to the formation of immunoglobulins. *KIT* encodes a receptor tyrosine kinase that is associated with the earliest neutrophil developmental stages [33]. *PTGS2* could activate the NF-κB signaling pathway which plays a key role in regulating the immune response to infection [34].

*Simultaneously, 2, 1, 1 and 2* candidate genes were opted for the concentration of IgG, IgG1, IgG2 and IgM in serum, respectively. Of these, *BAX* belongs to the BCL2 protein family which could regulate B cell homeostatic proliferation and apoptotic process [35]. *GRB2* encodes growth factor receptor-bound protein 2, which could regulate B-cell maturation, B-cell memory responses and inhibits B-cell Ca2^+^ signaling [36]. Thousand and one kinase 1 (TAOK1) could as a negative regulator of IL-17 to mediate signal transduction and inflammation, controlling colitis of inflammatory bowel disease [37]. *ICAM1* encodes a cell surface glycoprotein which is typically expressed on endothelial cells and cells of the immune system. Upregulation of ICAM1 in a mechanism involving NF-қB could inhibit the Epstein-Barr virus infection [38]. The expression level of TGFB1 was associated with melanoma immune response [39]. The protein encodes by *RAC3* is a member of the p160 family of nuclear receptor coactivators that plays an important role in NF-kappaB activation [40].

Generally, all these genes played vital roles in the inflammation, neutrophil activation, resistance to viruses, NF-kappaB, B cell homeostasis and immune-related process, which indicated the potentially important roles of Ig in colostrum and serum in resistance to infectious diseases.

In the present study we identified 8 and 6 first-time candidate genes for immunoglobulins in dairy cattle colostrum and serum, respectively. From the breeding perspective, our findings provide important molecular information for the genetic improvement program on health and disease-resistance traits in dairy cattle. On the other hand, as the absence of biological validation and the measurements of serum Ig in the offspring, further in-depth investigations are needed to better understand the genetic mechanisms on how these genes regulated and impacted the formation of immunoglobulins in colostrum before applying them on the breeding of dairy cattle.

## Conclusion

In this study, we conducted genome-wide association studies for the concentrations of immunoglobulins in colostrum and serum in Chinese Holstein. A total of 36 genome-wide and 82 suggestive significant SNPs were detected for the total IgG, IgG1, IgG2, IgA and IgM traits, in which the main quantitative trait loci for immunoglobulins were on BTA6 and 21. Combining the identified significant SNPs, functional enrichment, and the known QTL data, we identified 14 promising candidate genes for the concentration of IgG and IgM in colostrum and serum, including *FGFR4*, *FGFR2*, *NCF1*, *IKBKG*, *SORBS3*, *IGHV1S18*, *KIT*, *PTGS2*, *BAX*, *GRB2*, *TAOK1*, *ICAM1*, *TGFB1* and *RAC3*. Our findings provided new insights into the genetic architectures underlying immunoglobulins concentrations in colostrum and important molecular information for the genetic improvement program on these traits in dairy cattle.

## Methods

### Animals and phenotypes

The animals used in this study consist of 588 Chinese Holstein cows daughters of 44 sires from 10 dairy farms in the Beijing Dairy Cattle Center and the Beijing Sunlon Livestock Development Company Limited. The pedigree contained 1839 animals and was provided by the Beijing Dairy Cattle Center. The average number of daughters per sire was 13.4. Cows ranged from parity 1 to 4 (mean = 2.52). The blood serum and colostrum samples were taken from each cow during the first milking within 24 h after calving for measurement of immunoglobulins. Hair follicle samples were collected from each animal for SNP chip genotyping as well. The whole procedure for collection of the samples (blood, hair and colostrum) was implemented in strict accordance with the protocol approved by the Animal Welfare Committee of China Agricultural University (Permit number: DK996). The animals used in this study were all released to their own population for normal production after sample collection.

The concentrations of immunoglobulins of each colostrum and serum sample were measured, including total IgG (Bovine IgG ELISA Quantitation Set E10–118, Bethyl Laboratories, Montgomery, TX, USA), IgG1 (Bovine IgG1 ELISA Quantitation Set, E10–116), IgG2 (Bovine IgG2 ELISA Quantitation Set, E10–117), IgA (Bovine IgA ELISA Quantitation Set, E10–131) and IgM (Bovine IgM ELISA Quantitation Set, E10–101). For further statistical analysis, the phenotypic values for the concentrations of total IgG, IgG1 and IgG2 in colostrum or serum were square root transformed to fit a normal distribution, simultaneously phenotypes for IgA and IgM concentrations were log-transformed.

### Genotyping and quality control

Genomic DNA was extracted from the hair follicle samples with the QIAamp® DNA Mini Kit (QIAGEN, Valencia, CA, USA) for genotyping. A total of 588 individuals were genotyped with the GeneSeek GGP_HDv3 chip (150 K, including 140,668 SNP markers: GeneSeek, Lincoln, NE, USA).

Quality control was conducted on PLINK 1.90 software and the filtering processes were as follows: firstly, samples with all SNPs genotyping rate < 95% were deleted; then, SNPs with call rates < 90%, minor allele frequencies (MAF) < 0.1 and Hardy–Weinberg equilibrium (HWE) *p*-values < 10–6 were discarded [41, 42]. Thus, 563 individuals with 91,620 SNPs were kept for further analysis (Additional files 3 and 4).

### Statistical analysis

#### Mixed Model based single locus Regression Analyses (MMRA)

We performed single-SNP association analysis for the individual phenotype in GCTA 1.90.2 with the following mixed linear model:

$$\mathbf{y}=\mathbf{1}\boldsymbol{\upmu } +\mathbf{Xf}+b\mathbf{c}+\mathbf{Zg}+\mathbf{e}$$

Where **y** is a vector of transformed phenotypes (the concentration of IgG, IgG1, IgG2, IgA and IgM in colostrum and serum) of all cows; **μ** is the overall mean; **f** is the vector of fixed effects, including herd (classes: 1 to 10), parity (classes: 1 = parity 1, 2 = parity 2, 3 = parity 3 and 4 = parity 4) and season of calving (classes: 1 = March to May, 2 = June to August, 3 = September to November and 4 = December to February), **X** is an incidence matrix relating elements of **f** to **y**; **c** is the vector of the SNP genotype indicators which take values 0, 1 or 2 corresponding to the three genotypes 11, 12 and 22 (assuming 2 is the allele with a minor frequency), *b* is the regression coefficient of **y** on **c**; **g** is the vector of residual polygenic effects with **g ~ N** (0, **G**σ_g_^2^) (where **G** is the genomic relationship matrix and σ_g_^2^ is the additive variance), **Z** is the incidence matrix of **g**; **e** is the vector of residual errors with **e** ~ N (0, **I**σ_e_^2^) (where **I** is the indentity matrix and σ_e_^2^ is the residual variance). The heritability estimation were carried out by GCTA 1.90.2 software.

The existence of linkage disequilibrium (LD) of SNPs in every chromosome may lead to over-correction when using Bonferroni adjustments [41, 43]. Hence, we used an effectively independent test number to define the thresholds for genome-wide/suggestive significant associations based on the assessed number of independent markers and linkage disequilibrium blocks for markers on every chromosome [22].

Population stratification can result in spurious association findings in a GWAS [42]. Thus, we calculated the genomic inflation factor (λ) and depicted quantile-quantile (Q-Q) plot to assess stratification in our study population using qqman packages in R 3.6.0.

#### Identification of candidate genes

To further identify the candidate genes associated with the concentrations of immunoglobulins, we selected the functional genes that contained or were adjacent to the significant SNPs with less than 1 Mb based on the bovine gene set in RefSeq database (Bos_taurus_UMD_3.1; http://hgdownload.cse.ucsc.edu/goldenPath/bosTau6/database/). Additionally, to figure out the biological functions of these genes, Gene Ontology (GO) and Kyoto Encyclopedia of Genes and Genomes (KEGG) pathway enrichment were implemented with DAVID Bioinformatics Resources (https://david.ncifcrf.gov). In addition, we also compared the physical position of these functional genes with the reported quantitative traits loci (QTLs) for immune capacity traits in the Cattle QTL database (https://www.animalgenome.org/cgi-bin/QTLdb/BT/index).

## Supplementary Information

**Additional file 1: Table S1.** The features of genes contained or were near to (within 1 Mb) the identified significant SNPs for immunoglobulins.

**Additional file 2: Table S2.** Functional enrichment results of GO and Pathway analysis on the 1083 genes.

**Additional file 3.** MAP file of SNP data.

**Additional file 4.** PED file of SNP data.

## Data Availability

All supporting data can be found within the additional files.

## Abbreviations

Ig: Immunoglobulin
GWAS: Genome-wide association study
SNP: Single nucleotide polymorphisms
RUNX3: RUNX family transcription factor 3
BTA: *Bos taurus* autosome
MMRA: Mixed model based single locus regression analyses
GRM: Genetic relationship matrix
REML: Restricted maximum likelihood
LD: Linkage disequilibrium
QQ: Quantile-quantile
GO: Gene Ontology
KEGG: Kyoto Encyclopedia of Genes and Genomes
QTL: Quantitative trait locus
*FGFR4*: Fibroblast growth factor receptor 4
*FGFR2*: Fibroblast growth factor receptor 2
*NCF1*: Neutrophil cytosolic factor 1
*IKBKG*: Inhibitor of nuclear factor kappa B kinase regulatory subunit gamma
*SORBS3*: Sorbin and SH3 domain containing 3
*IGHV1S18*: Immunoglobulin heavy variable 4–59
*KIT*: KIT proto-oncogene receptor tyrosine kinase
*IGHV1S18*: Receptor tyrosine kinase
*PTGS2*: Prostaglandin-endoperoxide synthase 2
*BAX*: BCL2 associated X, apoptosis regulator
*GRB2*: Growth factor receptor bound protein 2
*TAOK1*: Thousand and one kinase 1
*ICAM1*: Intercellular adhesion molecule 1
*TGFB1*: Transforming growth factor beta 1
*RAC3*: Rac family small GTPase 3
IKK: Inhibitor of kappaB kinase
ROS: Reactive oxygen species
